# Validation of the International HIV Dementia Scale as a Screening Tool for HIV-Associated Neurocognitive Disorders in a German-Speaking HIV Outpatient Clinic

**DOI:** 10.1371/journal.pone.0168225

**Published:** 2016-12-19

**Authors:** Victor Marin-Webb, Heiko Jessen, Ute Kopp, Arne B. Jessen, Katrin Hahn

**Affiliations:** 1 Praxis Jessen^2^ + Kollegen, Berlin, Germany; 2 Department of Neurology, Charité - Universitätsmedizin Berlin, Berlin, Germany; The University of New South Wales, Neuroscience Research Australia, AUSTRALIA

## Abstract

**Background:**

HIV-associated neurocognitive disorders (HAND) are widely present among people living with HIV. Especially its milder forms, asymptomatic neurocognitive impairment (ANI) and mild neurocognitive disorder (MND), remain highly prevalent worldwide. Diagnosing these conditions is subject to a time and resource consuming neuropsychological assessment. Selecting patients at a higher risk of cognitive impairment by using a simple but effective screening tool helps to organise access to further neuropsychological diagnosis. The International HIV Dementia Scale (IHDS) has until now been a well-established screening tool in African and American countries, however these populations’ demographics defer significantly from ours, so using the same parameters could be ineffective.

**Objectives:**

To calculate the prevalence of this condition among people attending an HIV outpatient clinic in Berlin and to validate the use of the IHDS as a screening tool for HAND in a German-speaking population.

**Methods:**

We screened 480 HIV-infected patients using the IHDS, 89% of them were on a stable antiretroviral treatment. Ninety of them completed a standardised neuropsychological battery of tests and a specific cognitive complaints questionnaire. The same procedure was applied to a control group of 30 HIV-negative participants. HAND diagnosis was established according to the Frascati criteria.

**Results:**

The overall prevalence of HAND in our cohort was 43% (20% ANI, 17% MND and 6% HIV-associated dementia). The optimal cut-off on the IHDS for detecting HAND cases was set at 11 and achieved both a sensitivity and a specificity of 80%. When specifically screening for the more severe form of HAND, HIV-associated dementia, a cut-off value of 10 offered an increase in both sensitivity (94%) and specificity (86%). The Youden Index for diagnostic accuracy was 0.6 and 0.8, respectively.

**Conclusions:**

The prevalence of HAND was comparable to the reported by recent studies performed in countries with a similar economic development. The study confirms the IHDS to be a useful HAND screening tool in primary care settings and establishes new recommendations for its use in German-speaking countries.

## Introduction

HIV-associated neurocognitive disorders (HAND) are common among people living with HIV. Several studies have estimated the prevalence to range between 20% and 50% despite effective antiretroviral therapy [1, 2]. This may mean that up to half the people living with HIV worldwide –around 35 million according to UNAIDS [3]–are at risk of developing some degree of associated cognitive disorder, including HIV dementia. Neurocognitive dysfunction can limit social and work interactions, reduce the patient’s adherence to antiretroviral treatment, increase the risk of other conditions and ultimately lead to a deterioration in quality of life [4]. The latest diagnostic standards, published by Antinori et al. in 2007 and commonly known as the Frascati criteria, divide HAND into three distinct entities: asymptomatic neurocognitive impairment (ANI), mild neurocognitive disorder (MND) and HIV-associated dementia (HAD). These conditions are based on three parameters: performance during neuropsychological testing, existence or not of functional decline and no evidence of any other condition that could explain the symptoms [5]. The need for a complete neuropsychological assessment represents the biggest limitation for diagnosing HAND in primary and secondary care HIV clinics, as it is extremely time and resource consuming and needs to be performed by a trained neuropsychologist. A systematic neuropsychological testing of all HIV positive patients is difficult to implement and not suitable for administration in most primary and secondary care clinics. From the authors’ point of view, there is a lack of locally validated diagnostic screening tools.

The first published screening tool for HIV-associated dementia was the HIV Dementia Scale in 1994 [6]. Since then, several other screening tools have been proposed [7–11]. To date, there has been no consensus about which screening tool offers the best reliability, as not all screening tools apply equally to all populations, clinical settings and budgets. The International HIV Dementia Scale (IHDS) [8] was designed to be a brief, easy to administer, cross-cultural screening tool to identify individuals at risk of HIV dementia worldwide. We decided to use this tool for our study because it is rapid to administer -under five minutes-, it is language and culturally neutral and can be performed by any physician without specific neurological training. These three criteria are appropriate for the patients visiting our primary care clinic, which includes a high proportion of international clients.

To our knowledge, none of these screening tools have been administered to a large group of patients in any German-speaking country. We have therefore relied on similar results of international studies with foreign populations. This study aims to: Firstly, validate the clinical use of the IHDS as a screening tool for HAND in a German-speaking population and to establish local norms of use; Secondly, to calculate the prevalence of this condition among people attending an HIV outpatient clinic in Berlin, Germany.

## Methods

### Screening tools and neuropsychological testing

Neurocognitive function was screened for using Sacktor’s IHDS [8]. This test consists of three parts, each one analysing a specific cognitive domain –motor speed, psychomotor speed and memory– and scoring up to a maximum of four points. The final score is the sum of the three sub-scores with a range from 0 to 12 points.

To measure functional decline we used a questionnaire developed by the German NeuroAIDS Association (DNAA) consisting of twenty-two yes-no questions intended to evaluate neurological abnormalities such as memory and motor impairment, depression and similar problems in HIV-positive patients. A copy of the questionnaire is available as an additional material (S1 File). Nine of these questions have been mentioned as potentially helpful in describing mild interference with everyday functioning, and therefore useful in discerning between ANI and MND. The questionnaire also gives an idea of the patient’s mood status, which is useful in identifying a current depressive episode.

For the purpose of our study we put together a neuropsychological battery of tests with eleven different tests covering eight ability domains. Table 1 shows each test with its evaluated domain(s). Raw scores were normalised to Z-scores using demographic corrections for age and education. A more detailed description of the norms used can be found on the right column of the table under “references”. The time between screening and neuropsychological testing was three months or less. The patient’s general practitioner performed the IHDS as part of the regular visits to the health centre. A further physician trained in neuropsychology performed the neuropsychological evaluations.

**Table 1:** Content of the neuropsychological battery of tests.

*Test name*	*Evaluated neurocognitive areas or domains*	*References*
Rey Auditory Verbal Learning Test (RAVLT)	Learning and recall–(verbal) memory	[12] ^a^^e^
Rey Visual Design Learning Test (RVDLT)	Learning and recall–(visual) memory	[13] ^a^
Rey-Osterrieth Complex Figure Test (ROCF)	Sensory-perceptual abilities, (Visual) memory	[14] ^a^
Digit Span	Attention / working memory, (Short-term) memory	[15] ^a^^e^
Horn’s Performance Test System, Subtest 3 (LPS-UT3)	Logical thinking / Non-verbal intelligence*	[16] ^a^^e^
d2 Test of Attention—Revised Version	Attention / working memory	[17] ^a^^e^
Colour-Word-Interference Test	Executive function	[18] ^a^^e^
Controlled Oral Word Association Test (COWA), Subtests S-Words and Subtest Animals	Language / verbal fluency	[19] ^a^^e^
Trail Making Test (TMT), Part A	Psychomotor speed	[20] ^a^^e^
Trail Making Test (TMT), Part B	Attention / working memory, Executive function	[20] ^a^^e^
Wechsler Adult Intelligence Scale (WAIS-III), Subtest Digit Symbol-Coding	Psychomotor speed	[21] ^a^^e^
Grooved Pegboard	Motor skills	[22] ^a^^e^^g^

* This domain not included in the Frascati definition, but needed for evaluation of other tests;

^a^: adjusted for age;

^e^: adjusted for educational level;

^g^: adjusted for gender.

### Study population

Four hundred and eighty HIV-positive patients, aged 19–80, were screened for neurocognitive dysfunction and functional decline as described. They were then divided according to their IHDS score and distributed into three subsamples: The ‘poor performance’ subsample (n = 49, 10%) included participants with a score of 10 points or fewer, the ‘average performance’ subsample (n = 87, 18%) scored between 10.5 and 11 points, and the ‘high performance’ subsample (n = 344, 72%) scored either 11.5 or 12 points. Then, thirty members of each subsample were randomly selected to establish three study groups—with identical names as the subsamples—and to undergo neuropsychological examination. Participants had all been diagnosed with HIV for at least three months and spoke fluent German. Participants were excluded if they had a confounding neurological or psychiatric condition, cancer, an active opportunistic infection or were currently using mind-altering substances. A control group of thirty participants with a documented negative HIV test within the year preceding the evaluation and the same eligibility and exclusion criteria, was recruited in parallel.

The ethics committee of the Charité School of Medicine approved the study. The general terms of data protection and the Charité ‘Good Medical and Scientific Practice’ statutes were applied. All the study procedures were conducted in accordance with the 1964 Declaration of Helsinki (fourth revision). Participants who met the eligibility criteria were given detailed information about the study and provided with a written consent form. Only after signing this form were they finally recruited.

### Statistical analysis

Descriptive statistics were used to compare characteristics between groups of individuals. All normally distributed continuous variables were reported as means and standard deviation (SD). All non-normally distributed continuous variables were reported as medians with interquartile ranges (IQR). Associations of categorical variables between the different groups and analysed factors were assessed using the chi-square test. For normally distributed and non-normally distributed continuous variables with more than two samples, ANOVA and Kruskal–Wallis one-way analysis of variance tests were used, respectively. Mann-Whitney U Test was used for non-normally distributed variables with two samples. The relationship between two variables was evaluated by linear correlation analysis. The Pearson correlation coefficient was applied to samples with normal distribution. For nonparametric samples, Spearman’s rho coefficient was used. The intensity of the association between a categorical and a quantitative variable was assessed using Cohen’s d association index. All p-values were 2-tailed and considered significant at p < 0.05. The optimal cut-off point for the screening test was assessed by a Receiver Operating Characteristic (ROC) curve. The analyses were performed using IBM SPSS Statistics version 22.0. Sensitivity, specificity, predictive values and Youden’s J-Index were calculated using a Microsoft Excel spreadsheet provided by The Critical Appraisal Skills Programme (CASPe), which is available at redcaspe.org. All raw data used in this study has been made available for download as an additional material (S2 File).

## Results

### Group characteristics

This study took place between April 2012 and July 2014. The demographic and clinical characteristics of the four study groups are summarised in Table 2. The vast majority of participants were men (98.3%), with a median age of 43 (IQR 35–51). Over a third (37%) were non-native German speakers. Table 3 gives a further description of the samples’ ethno-linguistic background. The mean education -total years’ attendance at a teaching institution- was 16 (IQR 14–18). There were no significant differences in age or education between the groups. The median duration of the HIV-infection since diagnosis was 83 months. The participants with poor performance in the IHDS had lived with the virus longer (152 months) than those with average (89 months) or high (53 months) performance. The median CD4^+^ nadir was directly related to the score obtained in the IHDS: participants with a lower score had lower nadir rates (209 cells/μL) than those with a higher score (324 cells/μL). The current CD4^+^ cell count at the time of neuropsychological testing did not show any differences between groups. Most participants (89%) were on antiretroviral treatment. The proportion of treated participants was smaller in the high performance group: 77% versus 93% in the poor and 97% in the average performance groups. With a median of 7, the antiretroviral central nervous system (CNS) penetration effectiveness rank (CPE) did not vary significantly between groups. There were no differences between groups regarding the use of efavirenz or having a hepatitis C co-infection.

**Table 2:** Group characteristics, scores obtained in the Int. HIV Dementia Scale (IHDS) and answers given in DNAA Questionnaire (sorted by study group).

	*All groups*	*Poor Performance*	*Average Performance*	*High Performance*	*Control*	*P—Value*
*Demographics*						
Participants, n *(%)*	120 *(100%)*	30 *(25%)*	30 *(25%)*	30 *(25%)*	30 *(25%)*	
Female, n *(%)*	2 *(1*.*7%)*	0	0	1 *(3*.*3%)*	1 *(3*.*3%)*	0.565
Non-native German speakers, n *(%)*	44 *(37%)*	10 *(33%)*	12 *(40%)*	12 *(40%)*	10 *(33%)*	0.903
Age range	23–62	25–62	28–59	23–57	23–56	
Age, median *(interquartile range)*	43 *(35–51)*	45 *(39–51)*	46 *(38–54)*	41 *(33–49)*	40 *(31–49)*	0.129
Education, in years, median *(interquartile range)*	15 *(13–17)*	15 *(13–15)*	16 *(14–18)*	16 *(15–17)*	16 *(15–17)*	0.587
*HIV infection*						
Months since testing HIV-positive, median	83	142	89	53		**0.025**
Current CD4^+^ count, range	139–1252	173–1252	154–1246	139–1064		
Current CD4^+^ count, median	554	556	548	577		0.610
Historical CD4^+^ nadir, range	0–565	2–441	0–555	40–565		
Historical CD4^+^ nadir, median	274	209	282	324		**0.036**
*ART information*						
Patients receiving ART, n *(%)*	80 *(89%)*	28 *(93%)*	29 *(97%)*	23 *(77%)*		**0.031**
Current CPE, range	3–12	3–12	4–10	7–9		
Current CPE, mean *(SD)*	7.25 *(1*.*12)*	7.07 *(1*.*39)*	7.34 *(1*.*14)*	7.35 *(0*.*65)*		0.238
Treatment includes efavirenz, n *(%)*	7 *(7*.*7%)*	2 *(6*.*6%)*	2 *(6*.*6%)*	3 *(10%)*		0.856
Hepatitis C co-infection, n *(%)*	3 *(3*.*3%)*	1 *(3*.*3%)*	1 *(3*.*3%)*	1 *(3*.*3%)*		0.990
*IHDS subtest scores*						
Motor Speed subtest, mean score *(SD)*	3.79 *(0*.*48)*	3.3 *(0*.*70)*	3.87 *(0*.*34)*	4	4	
Psychomotor Speed subtest, mean score *(SD)*	3.62 *(0*.*66)*	2.83 *(0*.*79)*	3.63 *(0*.*49)*	4	4	
Memory (Recall) subtest, mean score *(SD)*	3.62 *(0*.*57)*	3.22 *(0*.*76)*	3.44 *(0*.*57)*	3.9 *(0*.*20)*	3.92 *(0*.*19)*	
Total Score, mean *(SD)*	11.04 *(1*.*17)*	9.38 *(1*.*01)*	10.94 *(0*.*17)*	11.93 *(0*.*17)*	11.9 *(0*.*18)*	
*DNAA answers*						
Reported concentration problems, n *(%)*	43 *(35*.*8%)*	27 *(90%)*	6 *(20%)*	8 *(26*.*7%)*	2 *(6*.*7%)*	**<0.001**
Reported motor problems, n *(%)*	13 *(10*.*8%)*	11 *(36*.*7%)*	0	1 *(3*.*3%)*	1 *(3*.*3%)*	**<0.001**
Reported sleeping problems, n *(%)*	37 *(30*.*8%)*	17 *(56*.*7%)*	5 *(16*.*7%)*	10 *(33*.*3%)*	5 *(17%)*	**0.002**

*Poor Performance*: Participants with IHDS scores of 10 or fewer; *Average Performance*: Participants with IHDS scores of 10.5 or 11; *High Performance*: Participants with IHDS scores of 11.5 or 12; *Control*: HIV negative participants with IHDS scores of 11.5 or 12. SD: standard deviation; ART: antiretroviral treatment; IHDS: International HIV Dementia Scale; DNAA: German NeuroAIDS Association.

**Table 3:** Participants’ ethno-linguistic distribution.

**Native German Speakers**			**76 *(63%)***
**Non-Native German Speakers**			**44 *(37%)***
Spanish			7
	*Castilian*	*3*	
	*Ecuador*	*2*	
	*Colombia*	*1*	
	*Honduras*	*1*	
English			6
	*USA*	*3*	
	*UK*	*2*	
	*Belize*	*1*	
Arabic			4
	*Egypt*	*2*	
	*Algeria*	*1*	
	*Lebanon*	*1*	
Croat			4
Nordic			4
	*Swedish*	*3*	
	*Danish*	*1*	
French			3
Italian			3
Hungarian			2
Polish			2
Catalan			1
Czech			1
Georgian			1
Hebrew			1
Indonesian			1
Kurdish			1
Portuguese *(Brazil)*			1
Russian			1
Turkish			1

Participants with a low IHDS score reported more concentration problems in the DNAA Questionnaire (90% in low, 20% in average and 26% in high performance groups). Reporting concentration problems also showed a negative effect on the obtained IHDS score (d = -1,46; p < 0.001). Participants who were HIV positive obtained lower scores in all three subtests of the IHDS compared to the negative controls. This relationship was strongest in the psychomotor subtest (z = -3.99; p < 0.001), followed by the memory (word recall) subtest (z = -3.34; p < 0.001) and the motor speed (finger tapping) subtest (z = -2.89; p = 0.004).

A bivariate correlation analysis was performed between the possible risk factors that may influence HAND and the screening and diagnostic outcome. We observed that older participants scored lower (r = 0.22; p = 0.015). This same correlation was also found with respect to the CD4^+^ cell count nadir (r = 0.25; p = 0.003). In addition, participants who were being treated with antiretrovirals had lower screening scores (r = 0.22; p = 0.037). Similarly, participants with a lower CD4^+^ nadir had higher HAND incidence (r = 0.28; p = 0.009). Education, measured as years attending a teaching institution, showed to correlate with the diagnostic outcome even after applying demographic corrections (r = 0.35; p < 0.001). No further relevant correlations were found with the current CD4^+^ cell count, the current viral load, the viral load zenith, gender, hepatitis C co-infection or the use of efavirenz.

**Fig 1:**
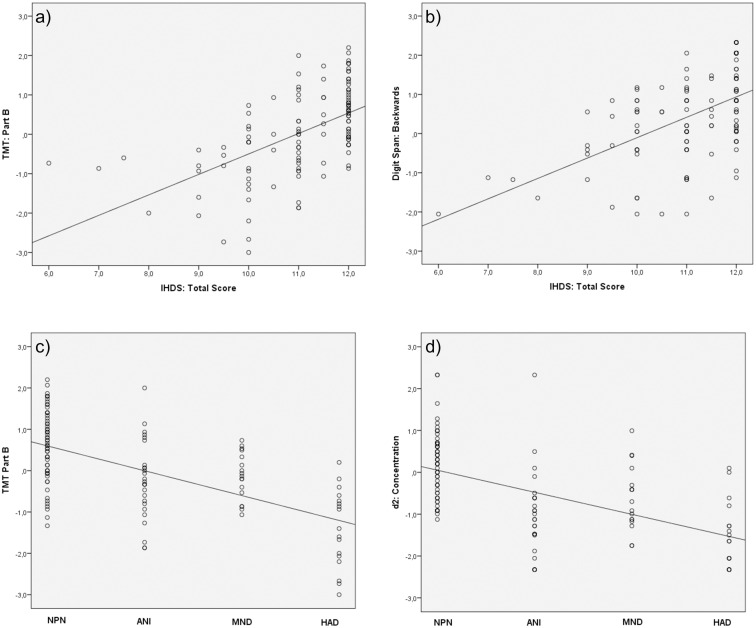
Correlation of the IHDS and a) TMT Part B and b) Digit Span Backwards subtests; and correlation of the HAND diagnostic subtype and c) TMT Part B and d) d2 Concentration subtests.

The IHDS had a moderate to strong correlation with individual neuropsychological tests. As shown in Fig 1, the highest correlation was found with the Trail Making Test (TMT) [20] Part B subtest (r = 0.552), followed by Digit Span Backwards [15] (r = 0.533), TMT Part A (r = 0.514) and d2-Test [17] (r = 0.499). Similarly, correlation findings were analysed by comparing individual tests and HAND diagnostic subtype. The best correlation was found with the TMT Part B (r = 0.602), followed by d2-Test (r = 0.599), TMT Part A (r = 0.506), Visual Learning [13] (r = 0.505) and ROCF immediate recall [14] (r = 0.500). All these correlations were significant at the p < 0.001 level.

### Measuring HAND prevalence

**Fig 2:**
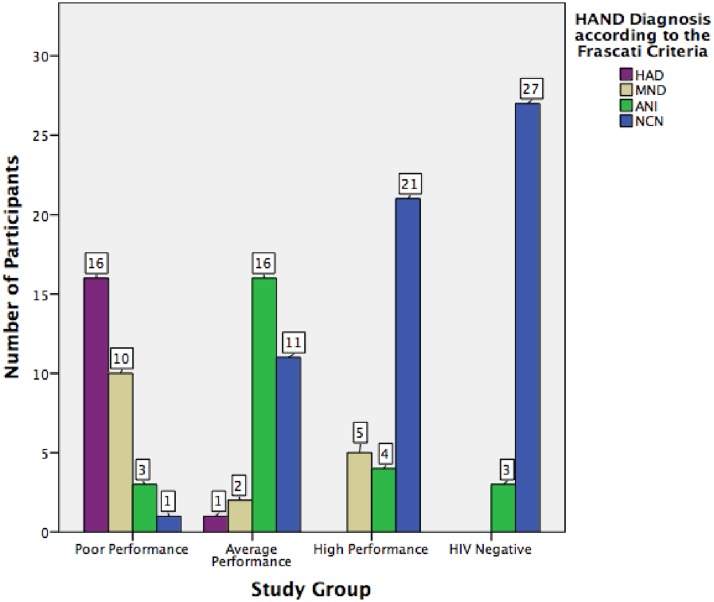
Number of cases of HAND and its subtypes in each study group.

The participants’ performance on the battery of neurocognitive tests and their subjective responses provided in the DNAA questionnaire were used to diagnose HAND according to the Frascati criteria [5] as follows: A person who obtained at least one negative standard deviation on a test would fail that test. People who failed two or more tests that evaluated at least two different cognitive domains were considered to have a HAND. Differentiation between ANI and MND was made depending on the answers given in the key questions of the DNAA questionnaire, which was used for evaluating cognitive decline. If the results obtained in the failed tests were of two negative standard deviations or more, and again these tests represented two different cognitive domains, the person was diagnosed with HAD. As shown in Fig 2, from the 90 evaluations performed, we found 23 cases of ANI, 17 of MND and 17 of HAD. The remaining 33 were neurocognitive normal (NCN).

The observed prevalence had to be adjusted to the size of the corresponding subsample. Therefore, we performed a weighted sum of the HAND cases in each group multiplied by their relative weight over the total population, using the following formula [23]:

$$\text{Adjusted Prevalence}=\frac{1}{\text{N}}\;\Sigma \text{i }\frac{\left({\text{e}}_{\text{i}}\;\cdot {\text{n}}_{\text{i}}\right)}{{\text{m}}_{\text{i}}}$$

where e_i_ = number of detected ANI, MND or HAD cases in one study group; n_i_ = total number of screened participants in one stratification group; m_i_ = size of study group (always 30); N = total size of the global screened population (always 480).

This way, the overall prevalence of HAND in our cohort was 43%. Of these, 47% represented ANI cases, 39% had MND and 14% were cases of HAD. The remaining 57% had a normal neuropsychological evaluation, and were considered to be NCN. In the control group, we found three cases (10%) with criteria compatible with ANI, and the rest were NCN (90%).

### Calculation of sensitivity, specificity and accuracy of the IHDS

Sensitivity and specificity of the IHDS were assessed using a ROC curve analysis. This statistical test allows us to determine the optimal cut-off value for the IHDS when screening for HAND by finding the point where both sensitivity and specificity are highest. To assess this calculation we used the data from all performed evaluations (90 in HIV positive plus 30 in HIV negative), being 60 (57+3) the total cases of HAND. The area under the curve was 0.843. We also calculated Youden’s Index for diagnostic accuracy, or J-Index, which captures the performance of a diagnostic test as a single number. This is defined by the formula *J = Sensitivity + Specificity − 1*, and its value ranges from 0 (meaning the test is useless), to 1 (test is perfect). Table 4, section a) shows sensitivity, specificity, predictive values and J-Index for the different cut-offs when looking for HAND in general, and section b) the same procedure, for HAD cases only, to see if the cut-off value and the accuracy changed when looking for the more severe cases of HAND. Section c) uses the cut-off value of 11 for detecting all HAND cases, separately for patients receiving or not an antiretroviral treatment.

**Table 4:** Characteristics of the different cut-off values on the IHDS.

	*Cut-Off*	*Sensitivity*	*Specificity*	*PPV*	*NPV*	*J-Index*
*Raw Scores*	*a) for all HAND*					
	9.5	22%	100%	100%	56%	0.2
	10	48%	98%	97%	66%	0.5
	10.5	52%	95%	91%	66%	0.5
	11	80%	80%	80%	80%	0.6
	11.5	83%	68%	72%	80%	0.5
	12	100%	0%	50%	100%	0.0
	*b) for HAD only*					
	9.5	47%	95%	62%	92%	0.4
	10	94%	86%	53%	98%	0.8
	10.5	100%	84%	50%	100%	0.8
	11	100%	58%	28%	100%	0.6
	11.5	100%	50%	25%	100%	0.5
	12	100%	0%	14%	100%	0.0
*ART Status*	*c) for all HAND*					
On ART	11	85%	58%	81%	65%	0.4
No ART	11	67%	86%	67%	86%	0.5
*T-Scores*	*d) for all HAND*					
	38	60%	86%	82%	68%	0.5
	39	63%	83%	79%	69%	0.5
	40	68%	82%	79%	72%	0.5
	41	76%	80%	79%	78%	0.6
	42	78%	72%	73%	78%	0.5

IHDS: International HIV Dementia Scale; HAND: HIV-associated Neurocognitive Disorder; HAD: HIV-associated Dementia; PPV: positive predictive value; NPV: negative predictive value; ART: antiretroviral treatment.

The variables education, age and their interaction were entered into the normative model following an adaptation of the methodology reported by Rourke (2003) and Heaton (2004) [24–26]. This was done because of the well-known influences of these variables in the outcome of neuropsychological tests [27]. The first step in the development of the T-score was to normalise the test score distribution, converting raw scores to scaled scores having a mean of 10 and a standard deviation of 3. Scaled scores range from 1 to 12, and higher scores reflect better test performance.

Next, the scaled scores were regressed on the three independent demographic variables (age, education and their interaction). The residuals of each of these variables obtained in the regression procedure were used to calculate an accordingly weighted, predicted scaled score. The final IHDS T-scores were obtained by using the following formula:

$$\text{Tscore}=\left[\left(\;\frac{\text{IHDS Scaled Score}-\text{Predicted Scaled Score}}{\text{Standard Deviation}}\right)\times \;10\right]+50$$

Raw to scaled score conversions and demographically adjusted T-scores obtained from applying age and education corrections to scaled scores can be found as additional material (S3 File). Table 4, section d) summarises the characteristics of the different T-scores. Consistent with published recommendations, the T-score cut-off was set at 40 (≤ impaired; > unimpaired).

## Discussion

### Screening tool

Until now, there has not been any standardisation of an internationally used screening tool for HAND in a German speaking population. This study confirms that the IHDS gives good results with regards to accuracy as well as ease of use. In a ROC curve analysis using a cut-off score of 11 or below, the IHDS was 80% sensitive and 80% specific in detecting cases of HAND. Several meta-analyses that have reviewed this screening tool, have reported a lack of accuracy due to a low specificity when screening for the milder forms of HAND, ANI and MND [28–31]. In our case, the recorded specificity is slightly higher than in previous studies [8, 32–37], resulting in moderate to high levels of accuracy, with J-Indexes of 0.6 when screening for HAND in general and 0.8 when screening for HAD specifically. Certainly, there is a difference between Zipursky et al.’s [31] obtained pooled sensitivity of 0.62 calculated across all listed publications referring to this screening tool and the 0.80 obtained in our study. These differences are caused by the variety of existing socio-cultural frameworks among the evaluated populations and the diversity of countries in which the studies took place, which, in our opinion, justifies the local standardisation and validation of the screening tool for each unique social and cultural context. The IHDS’s sensitivity when screening for HAND was higher in the participants who were receiving an antiretroviral treatment than in those who were still ART-naive. This relation was reversed when comparing specificity. These results should be considered with precaution, as the group of ART-naive participants was reduced: 10 vs. 80 on ART, meaning that 89% of participants were on treatment. The use of demographically adjusted T-scores represent an improvement in sensitivity and specificity when compared to the original raw cut-off value of 10, and achieve similar rates of accuracy when compared to the proposed higher raw cut-off value of 11 points. However, the use of transformation tables adds an extra level of execution for the evaluating nurses and doctors. We agree that a more simple screening method would promote its applicability and feasibility in primary care clinics. Therefore, we decided to make the transformation tables available as an additional material (S3 File) to those who are interested, but we suggest the raw score of 11 as the cut-off point to be used in daily routine. We understand that using the raw score allows a simpler understanding and execution of the IHDS for physicians and nurses not trained in neuropsychology.

Despite not being a substitute for complete neuropsychological testing, the IHDS has shown to be effective in pointing out those patients at a higher likelihood of developing HAD, which allows healthcare providers to refer these patients to neuropsychological evaluation when needed.

Based on the results of our study, we suggest the following recommendations when using the IHDS in a German-speaking population:

1. All patients who score 11 or below should be neuropsychologically tested in order to exclude an ongoing HAND.
2. Those who score 10 or below should be evaluated without delay, as this indicates a higher likelihood of being diagnosed with HAD.
3. All patients who score over 11 points and actively express cognitive complaints should also be neuropsychologically tested in order to exclude ongoing HAND. Current major depression or psychiatric disturbance should be ruled out as a cause of the complaints.
4. All those who score over 11 points and do not express any cognitive complaints should be rescreened in six months.

Please note that an ‘abnormal’ screening result should not be interpreted as a conclusive diagnosis of dementia. The screening result could be influenced by other comorbid conditions, such as low mood, depression or substance use. Also, if advanced impairment is highly suspected, neurocognitive assessment should not be deferred in any case, regardless of the score obtained in the screening procedure.

Theoretically, the IHDS could be used for rescreening as well as for monitoring purposes of patients with an already diagnosed HAND. In order for the IHDS to track fluctuations in cognition, it is important to determine the test’s repeatability, intra-subject variation, and learning effects [30]. This study did not address this issue. A literature search provided a study that evaluated the test-retest reliability of the IHDS when performed twice on the same patient within a one-week interval [38]. The study showed a good test-retest correlation between the total score, the finger tapping and psychomotor tasks, however the correlation reduced in the memory recall task. Indeed, this study does not answer the question about a possible learning effect on the four-word task when retested. Nevertheless, a second version of a test for avoiding learning effects when retesting is available for a number of neuropsychological tests. From our point of view, it would be of major interest and utility to have further standardised sets of four words available for re-screening purposes.

The application of the IHDS in a primary care setting has been welcomed and positively evaluated both by patients and healthcare practitioners. The main limitation was the test’s inability to discern between HAND subtypes, especially between ANI and MND. Concerning our study, this was of lesser importance, as our aim in this very initial diagnostic stage was to see if the test was able to reveal which individuals were at risk of HAND in order to refer them for neurological evaluation, without the need for further characterising the deficit. Nevertheless, the ability of a screening tool to discern between ANI, MND and HAD remains a key area of discussion and debate in the NeuroAIDS field. The IHDS, as it is currently designed, does not allow evaluating cognitive decline, which hinders a HAND subclass differentiation. This could be easily completed in a future version of the screening tool by adding an item evaluating functional decline as a fourth point of evaluation (i.e. short questionnaire or direct questions from the examiner). As an example of the latter, we conducted an analysis that individually crossed the results of the IHDS and the neuropsychological assessment with the DNAA questionnaire in order compare the HAND outcome depending on the neuropsychological tool used. 53% (48/90) had been marked as impaired in both tools; 23% (21/90) as unimpaired in both tools; and 23% (12+9/90) had divergent results between tools. The correlation coefficient between the results obtained in IHDS and neurocognitive assessment when comparing impaired vs. unimpaired was r = 0.49 (p < 0.001). With a 23% of inaccuracy of the IHDS when used for diagnosing HAND, this analysis shows that the use of this tool as a substitute for the neuropsychological examination is not recommended.

### Prevalence

The prevalence of HAND varies substantially depending on the consulted source [1, 39–43]. In recent literature, prevalence ranged from 69% in a wider cohort of people living with HIV in French-speaking Switzerland [41] to 21% in a more specific cohort of urban men who have sex with men in the London metropolitan area [39]. Several reasons explain this fluctuation: Population differences existing between the different study groups -age, gender, educational level, comorbidities and viral control- play a determinant role [9]. In other cases, the wide use of standardised norms in populations being falsely considered equal has brought upon misclassification of patients, thereby altering the proportions of affected individuals and the global prevalence in the cohort, as a study by Cysique et al. shows, comparing US and Australian populations [40]. This supports the idea of developing local normative standards for tests and screenings that apply to specific linguistic, social and cultural groups of people.

This study shows that HAND prevalence remains high regardless of the close medical surveillance that the patients in the Berlin cohort receive. Moreover, this study reveals a large proportion of subjective cognitive complaints as well as neuropsychological deficits despite the wide use of antiretrovirals with an assumed appropriate CNS penetration index. As mentioned in Table 2, 89% of participants were receiving antiretroviral treatment and the mean CPE score was found to be higher than seven for all study groups. When asked if they were currently experiencing cognitive complaints, 35.8% of the study population reported having experienced some in the last three months. This proportion rose to 90% in the ‘poor performance’ group. This shows us which population we should focus on more: those actively expressing cognitive complaints. More work should be performed on this group of patients in order to better understand the high level of complaints and also to discard possible viral escape situations.

The wider analyses of the data from our cohort brought up several other key factors that potentially could lead to an early detection of the disorder. Education proved to be an important predictor of HAND diagnosis. The fewer the years spent in education, the greater the proportion of HAND cases, especially in its more severe forms. Advanced age also appeared to be a significant factor in developing this condition. Both formal education and age have been reported to have an influence in the outcome of neuropsychological tests. Even after applying demographic corrections for these two factors, education and age showed to have some degree of correlation with HAND. These finding have also been reported by other study groups in the past [44, 45]. By contrast, having a non-German linguistic background did not show any specific effect on the neuropsychological outcome. Even if the statistical significance of a variable with such wide standard deviations is questionable, in this cohort, participants with a lower CD4^+^ nadir had higher HAND incidence. CD4^+^ nadir has been considered to have an association with cognitive impairment in HIV-infected individuals by many authors [46, 47]. A state of extreme immune suppression with very low CD4^+^ cell counts is likely to produce irreversible neural injury [48]. Combined with the certainty that ongoing replication in the CNS despite controlled systemic viral suppression causes cognitive difficulties [49], these two arguments support an early start of antiretroviral treatment in all HIV positive patients to reduce the risk of HAND.

Currently there is no consensus as to which tests should be part of a common neuropsychological battery of tests for detecting HAND. From this study we learn that certain tests correlate better with the diagnosis of HAND than others, especially the TMT Part A and B tests, the d2 concentration test and the Digit Span test. Testing attention/working memory and information processing speed has been described as particularly useful in detecting neurocognitive impairment in people living with HIV [50]. These tests should be part of any neuropsychological battery of tests for detecting HAND, given their high predictive power as well as easy performance and interpretation.

We wanted to better understand the results from those participants who expressed no cognitive complaints, as almost the half (47%) of the HAND diagnosed cases were asymptomatic. The significance of ANI has been questioned in the NeuroAIDS field since the publication of the Frascati definition. The real implications of ANI have been criticised by some authors, implying that, as it is now defined, it falsely inflates the prevalence rate [39]. In fact, 10% (3) of our healthy controls had criteria of ANI after completing cognitive testing. Some authors have suggested raising the impairment threshold in neuropsychological tests in order to reduce false positive rates, which are calculated to be as high as 21% [51]. In contrast, other newly published studies have added reliable data that supports the clear prognostic significance of a diagnosed ANI. A study by Grant et al. showed that being diagnosed with ANI increased the risk of suffering MND or HAD two to six-fold [52]. A further neuropsychological evaluation of the patients in our cohort, after some time, can clarify this, as it would record any progression -or regression- in their HAND stage.

This study has several limitations: it is a single centre study, with a relatively small sample size and a limited gender profile –in our case almost exclusively men who have sex with men–. Additionally, there are individual factors of the participants that work as diagnostic confounders—such as past episodes of depression, long-term unemployment and prior use of alcohol and substances. As already mentioned, a learning effect on the four-word task of the IHDS may appear when re-testing. This is a known event in neuropsychological testing that can be easily solved by designing different versions of the same task, which must be similarly empirically validated prior to its clinical use. If this task is to be utilised in a longitudinal context, future studies are required to clearly understand the role of practice effects, test-retest reliability, and regression to the mean on test scores in this local population. 70% of the screened population obtained an almost perfect IHDS score. This suggests a ceiling effect that could question the utility of the test. A possible explanation could be the educational makeup of the sample, with a high proportion of university-educated participants. However, the use of the IHDS has managed to refer patients without apparent clinical abnormalities to neuropsychological assessment, which confirmed an asymptomatic neurocognitive disorder. In addition, its use has brought awareness of a condition that patients and primary care doctors were previously not familiarised with, as they now openly speak about it. We find these achievements to be of major importance. Cognitive complaints were evaluated by a single, self-reporting questionnaire. All answers were given as dichotomic, which could have limited the information obtained, although its use is widely spread in research. Also, when applied to a group of patients with advanced impairment, self-reported questionnaires may lack the ability to provide an accurate response, as these patients may be unaware of their own decline. Their answers can mask their actual symptoms, and can result in a misclassification of that patient as asymptomatic. Finally, the size of our neuropsychological battery of tests was relatively larger than that used by other study groups, increasing the likelihood of obtaining a lower score in two or more domains.

## Conclusions

This study reports that HAND are still widely present among people living with HIV. Even when having the widest options of antiretroviral medication and treatments available, the prevalence of HAND in the Berlin cohort remained high: 43%, or 20% ANI, 17% MND and 6% HAD. Screening for HAND is an interesting approach in primary care. A regular screening can detect early impairment and defines a cognitive trend even before the patient starts expressing subjective complaints. It should be an essential part of any new HIV diagnosis and become part of regular medical check-ups in chronically infected patients in order to improve the general prognosis of the condition. The IHDS has been proven to be an inexpensive, rapid and easy to administer screening tool for HAND. It is effective in discerning between patients with possible neurocognitive impairment and those with normal neurocognitive function. This study has developed a new local normative standard with new cut-off values and proceeding recommendations valid for German-speaking populations.

## Supporting Information

S1 File DNAA Questionnaire, Questionnaire used to evaluate neurological abnormalities. (PDF)

S2 File Study Data, Raw data used for the analyses. (ZIP)

S3 File Adjusted Normative Tables, Raw to scaled score conversions and demographically adjusted T-scores tables. (PDF)
