# Supplementary material for: Validation of the International HIV Dementia Scale as a Screening Tool for HIV-Associated Neurocognitive Disorders in a German-Speaking HIV Outpatient Clinic
Source: PLoS One. 2016 Dec 19;11(12):e0168225. doi: 10.1371/journal.pone.0168225 (PMC5167352; doi:10.1371/journal.pone.0168225)
Supplement: S1 File — (PDF) [file pone.0168225.s001.pdf]

ADDITIONAL MATERIAL

Questionnaire of the DNAA – Page 1

SNAS – Short Neuro-AIDS Screening - German NeuroAIDS Study Group

DNAA Deutsche Neuro-AIDS Arbeitsgemeinschaft e.V., http://www.dnaa.de

NeuroScreening

für Neurokognitive Defizite in HIV+ Patienten

Patientennummer:	*Geschlecht:* ☐ *M* ☐ *W* *Alter:*
Können Sie seit mehr als 3 Monaten nicht mehr konzentriert den gewohnten Arbeiten und Freizeit-Tätigkeiten nachgehen? ☐ *Ja* ☐ *Nein*	*Fällt Ihnen mehrmals am Tag ein Gegenstand aus der Hand?* ☐ *Ja* ☐ *Nein*
Müssen Sie die Seiten eines Buches oder einer Zeitung mehrfach lesen, um den Inhalt zu behalten? ☐ *Ja* ☐ *Nein*	*Sind Sie seit mehr als 3 Monaten depressiv gestimmt, ohne dass große Veränderungen in Ihrem Leben aufgetreten sind?* ☐ *Ja* ☐ *Nein*
Hat sich Ihr Schriftbild verändert? ☐ *Ja* ☐ *Nein*	*Sind Sie tagsüber ständig müde, obwohl Sie nachts schlafen?* ☐ *Ja* ☐ *Nein*
Können Sie kleine Schrauben nicht mehr andrehen, keine Knöpfe mehr annähen oder sind Sie am Computer langsamer geworden? ☐ *Ja* ☐ *Nein*	*Haben Sie mehr als 3 Monate andauernde Ein- und/oder Durchschlafstörungen?* ☐ *Ja* ☐ *Nein*

	Ja	*Nein*
Spricht Ihr persönliches Umfeld Sie darauf an, dass Sie sich verändert haben?		
Waren Sie in den letzten 12 Monaten mehrfach krankgeschrieben, ohne dass eine internistische/allgemeinmedizinische Erkrankung zugrunde lag (allgemeiner Erschöpfung)?		
Sind Sie älter als 65 Jahre?		
Sind Sie bereits seit dem Kindes-/Jugendalter mit HIV infiziert?		
Befinden Sie sich zur Zeit in einer Therapiepause?		
Haben Sie eine antiretrovirale Therapie bei einer geringen CD4-Zellzahl (<250Zellen/ml) begonnen?		
Gibt es eine nachweisbare Viruslast trotz der Therapie?		
Verspüren Sie ungewöhnliche Kribbel-/Wärme-/Kältegefühle, Schmerzen oder Taubheit in den Händen und Füßen?		
Stolpern Sie häufig?		
Bemerken Sie einen Rückgang der Muskulatur, nicht gut heilende Wunden oder Verfärbungen der Haut an den Füßen und Unterschenkeln?		
Haben Sie Potenz- bzw. Libidostörungen?		
Bemerken Sie Störungen beim Wasserlassen?		
Haben Sie Videx, Zerit oder Tuberkulosemittel in den letzten 12 Monaten genommen?		
Leiden Sie unter Diabetes mellitus (Zuckerkrankheit)?		

HCV/HBV Serologien	*Vorerkrankungen*	*Alkoholkonsum*	*Konsum tox. Substanzen*

Note: The design and content have been adapted to the needs of our study.

Questionnaire of the DNAA – Page 2

SNAS – Short Neuro-AIDS Screening - German NeuroAIDS Study Group

DNAA Deutsche Neuro-AIDS Arbeitsgemeinschaft e.V., http://www.dnaa.de

**Behandler Teil**

HIV ED:	*Infektionszeitpunkt:*

Aktuelle ART: ☐

Kommentare
Konzentration
Gedächtnis
Feinmotorik
Stimmung
Schlaf
PNP

International HIV Dementia Scale – IHDS		*Laborwerte*	
MS		*Viruslast-PCR*	
PMS		*T-Helferzellen abs*	
MR		*T-Helferzellen rel*	
Punktzahl	/12	*Ratio CD4/CD8*	
☐*Unauffällig*		*Viruslast Zenit*	
☐*Grenzwertig*		*T-Helferzellen Nadir*	
☐*Auffällig, eine Weitertestung wird empfohlen.*		*TPPA / VDRL*	

Note: The design and content have been adapted to the needs of our study.
