# Supplementary material for: Validation of the International HIV Dementia Scale as a Screening Tool for HIV-Associated Neurocognitive Disorders in a German-Speaking HIV Outpatient Clinic
Source: PLoS One. 2016 Dec 19;11(12):e0168225. doi: 10.1371/journal.pone.0168225 (PMC5167352; doi:10.1371/journal.pone.0168225)
Supplement: S3 File — (PDF) [file pone.0168225.s003.pdf]

PONE-D-16-07464

"Validation of the International HIV Dementia Scale as a screening tool for HIV-Associated Neurocognitive Disorders in a German-speaking HIV outpatient clinic"

Normative conversion of raw IHDS total scores to Scaled Scores	
Raw Score	Scaled Score
≤6	1
6.5	2
7	3
7.5	4
8	5
9	6
9.5	7
10	8
10.5	9
11	10
11.5	11
12	12

PONE-D-16-07464

“Validation of the International HIV Dementia Scale as a screening tool for HIV-Associated Neurocognitive Disorders in a German-speaking HIV outpatient clinic”

Demographically-adjusted normative standards (T-scores, M=50; SD=10)

Education 8 -12 Years

	Age								
SS	21-25	26-30	31-35	36-40	41-45	46-50	51-55	56-60	61-65
1	0	0	0	0	0	1	2	3	4
2	0	2	3	4	5	6	7	8	9
3	5	6	7	8	10	11	12	13	14
4	10	11	12	13	14	15	16	18	19
5	15	16	17	18	19	20	21	22	23
6	20	21	22	23	24	25	26	27	28
7	24	25	26	28	29	30	31	32	33
8	29	30	31	32	33	34	35	37	38
9	34	35	36	37	38	39	40	41	42
10	39	40	41	42	43	44	45	46	47
11	43	44	45	47	48	49	50	51	52
12	48	49	50	51	52	53	55	56	57

PONE-D-16-07464

"Validation of the International HIV Dementia Scale as a screening tool for HIV-Associated Neurocognitive Disorders in a German-speaking HIV outpatient clinic"

Demographically-adjusted normative standards (T-scores, M=50; SD=10)

Education 13 - 15 Years

	Age								
SS	21-25	26-30	31-35	36-40	41-45	46-50	51-55	56-60	61-65
1	0	0	0	0	0	0	0	1	2
2	0	0	0	1	2	3	4	6	7
3	2	3	5	6	7	8	9	10	12
4	7	8	9	10	12	13	14	15	16
5	12	13	14	15	16	18	19	20	21
6	17	18	19	20	21	22	24	25	26
7	21	22	24	25	26	27	28	29	31
8	26	27	28	30	31	32	33	34	35
9	31	32	33	34	35	37	38	39	40
10	36	37	38	39	40	41	43	44	45
11	40	41	43	44	45	46	47	48	50
12	45	46	47	49	50	51	52	53	54

PONE-D-16-07464

"Validation of the International HIV Dementia Scale as a screening tool for HIV-Associated Neurocognitive Disorders in a German-speaking HIV outpatient clinic"

Demographically-adjusted normative standards (T-scores, M=50; SD=10)

Education 15 - 20 Years

	Age								
SS	21-25	26-30	31-35	36-40	41-45	46-50	51-55	56-60	61-65
1	0	0	0	0	0	0	0	1	1
2	0	0	0	0	0	1	2	3	5
3	0	0	2	3	4	6	7	8	9
4	4	5	6	8	9	10	12	13	14
5	9	10	11	13	14	15	16	18	19
6	13	15	16	17	19	20	21	22	24
7	18	20	21	22	23	25	26	27	28
8	23	24	26	27	28	29	31	32	33
9	28	29	30	32	33	34	35	37	38
10	33	34	35	36	38	39	40	41	43
11	37	39	40	41	42	44	45	46	47
12	42	43	45	46	47	48	50	51	52
